# Localized measurement of ultrasonic waves using a Fabry–Perot sensor illuminated by a Bessel beam

**DOI:** 10.1364/AO.548048

**Published:** 2025-03-19

**Authors:** Dylan M. Marques, Oliver Sheppard, Edward Z. Zhang, Peter R. T. Munro, James A. Guggenheim

**Affiliations:** 1 Department of Electronic Electrical and Systems Engineering, School of Engineering, University of Birmingham, Birmingham, UK; 2 Department of Cardiovascular Sciences, School of Medical Sciences, University of Birmingham, Birmingham, UK; 3 Department of Medical Physics and Biomedical Engineering, University College London, London, UK; 4 Wellcome/EPSRC Centre for Interventional and Surgical Sciences, University College London, London, UK

## Abstract

Fabry–Perot (FP) ultrasound sensors are a class of optical ultrasound sensors used in photoacoustic tomography (PAT) and other applications. Conventionally, an FP ultrasound sensor comprises an ultrasonically compressible planar microcavity, locally interrogated by a focused Gaussian beam. One way to increase the sensitivity could be to replace this beam with a Bessel beam. The rationale is twofold. First, as a Bessel beam’s wavefront better matches the modes of the planar microcavity, this could increase the Q -factor, leading to higher sensitivity. Second, as a Bessel beam provides a focused spatial structure—a central core surrounded by concentric rings—it might retain the ability to locally interrogate the sensor. To explore this idea, we developed an experimental system featuring a custom FP ultrasound sensor interrogated by a Bessel beam and evaluated its on-axis sensitivity, directivity, and image resolution when performing PAT. For comparison, we measured the same characteristics using a conventional focused Gaussian beam with a spot size similar to the core of the Bessel beam. As anticipated, the Bessel beam provided a higher ultrasonic on-axis sensitivity. However, the directivity and spatial resolution were degraded, suggesting that the Bessel beam yielded a larger acoustic element. We conclude that it is feasible to increase the sensitivity of an FP ultrasound sensor using a Bessel beam. Further work is required to establish whether differently designed Bessel beams could concurrently offer a smaller acoustic element.

## 1. INTRODUCTION

Fabry–Perot (FP) ultrasound sensors are a class of ultrasound sensors based on compressible optical microcavities. They are used in a range of areas, including photoacoustic tomography (PAT) [1], photoacoustic microscopy [2], pulse-echo ultrasound [3], and ultrasonic metrology [4]. In PAT, FP sensors have been widely demonstrated to provide images with high fidelity and spatial resolution [1,5–7]. This imaging performance arises due to the broadband frequency response, small acoustic element size, and high sensitivity of the FP sensor. The small element size, for instance, allows for the omnidirectional detection of ultrasonic waves and the high spatial sampling required for high-resolution imaging [8]. Meanwhile, the high sensitivity enables the detection of weak ultrasound waves generated deep inside tissue [9]. Further increasing this sensitivity is of interest for a range of reasons. For example, by allowing the detection of weaker ultrasound waves, it could enable imaging pathologies deeper in tissue, thereby broadening the applicability of PAT [10].

FP ultrasound sensors typically comprise a transparent planar polymer layer situated between two highly reflective mirrors. When illuminated by a focused interrogation laser beam, the sensor acts as an optical cavity [2,11,12]. The laser light resonates inside the FP cavity if its wavelength closely matches a half-integer fraction of the thickness of the optical cavity [13]. Outside resonance, FP sensors have high reflectance, but the reflectance sharply reduces as the resonance condition is approached. Consequently, the wavelength-resolved reflectance of FP sensors, referred to as the interferometer transfer function (ITF), comprises one or multiple sharp interference fringes [13]. To detect ultrasound, the wavelength of light is chosen to be at the edge of one of the fringes, near the peak gradient of the ITF. When interrogated at this wavelength, ultrasonically induced modulations of the cavity thickness are mapped into the FP sensors’ reflectance, which can be monitored using a photodiode [14]. Altogether, this provides a means to optically measure ultrasound waves.

One way to increase the sensitivity could be to increase the sharpness of the ITF by increasing the Q -factor of the cavity [15]. In principle, increasing the Q -factor can be achieved by increasing either the mirror reflectivity or the cavity thickness [16]. However, beyond a certain point, the Q -factor becomes limited by the matching of the interrogation beam to the FP cavity modes. To provide a good match to a cavity mode, the wavefront and profile of the interrogation beam must self-replicate efficiently during its propagation back-and-forth inside the cavity [17]. In a planar cavity, the modes are any beams comprising optical waves presenting one, and only one, angle of incidence to the optical axis [18,19]. As a focused Gaussian beam comprises a broad angular spectrum of plane waves presenting many angles to the optical axis, it is a poor match to the modes of a planar cavity [20]. In some cases, the results of this mismatch are barely noticeable. For example, in few-tens-of-microns-thick FP sensors with mirrors of less than about 95% reflectivity, the Q -factor of an FP sensor interrogated by a 50 µm Gaussian beam can readily approach the theoretical limit predicted for a perfectly matched plane wave [16]. In other cases, however, namely when the mirror reflectivity or thickness is increased enough to offer a significantly higher Q -factor, the mismatch leads to a broadening of the fringes of the ITF and a reduction in the fringe visibility, reducing the gradient of the ITF, and thus limiting the optical sensitivity [16].

One way to overcome the limitations imposed by the mismatch of a planar cavity with a Gaussian beam is to design FP sensors with a different geometry. For example, Guggenheim *et al.* developed FP ultrasound sensors with a planoconcave cavity formed by one planar and one curved mirror [15], and Thathachary and Ashkenazi incorporated waveguides inside the FP cavity [21]. In both cases, the change in the FP geometry led to a focused Gaussian beam matching a mode of the cavity, enabling higher sensitivity. Although promising, these approaches introduce additional challenges related to sensor fabrication and interrogation. For example, unlike how a Gaussian interrogation beam can be freely placed at any location upon a planar FP sensor, it must be positioned precisely at locations defined only by the center of the curved mirrors or embedded waveguides. This makes it more challenging to interrogate these sensors, particularly using multi-beam readout systems required for high-speed imaging [22].

Aside from changing the cavity geometry, another approach could be to use a different interrogation beam that better matches the mode of the cavity while still affording a small acoustic element size. A promising candidate is a Bessel beam, a type of beam comprising a core surrounded by concentric rings, formed by interfering a continuum of plane waves propagating at a common angle to an optical axis [23]. The rationale is twofold. First, as the beam is composed of plane waves at a single angle, it could provide better mode-matching with the planar cavity, unlocking a higher Q -factor, and thus a higher ultrasonic sensitivity. Second, as the profile features a focused core surrounded by rings, it could also retain the ability to provide a small acoustic element size, as required for high-resolution imaging.

Recently, experiments demonstrated that the maximum ITF gradient of a fused silica FP etalon could be increased by two times when replacing a 50 µm Gaussian interrogation beam with a Bessel beam with a 30 µm core. However, the approach has not yet been applied to interrogate an FP ultrasound sensor. As such, testing whether such an interrogation scheme can be used to increase the ultrasonic sensitivity and retain a small acoustic element size represents an open challenge.

To address this challenge, we designed and constructed an ultrasonic field mapping system based on a custom FP ultrasound sensor interrogated using a Bessel beam. To test its ultrasonic characteristics, we measured ultrasonic sensitivity, directivity, and spatial resolution when performing PAT. The same tests were performed using conventional Gaussian beam interrogation and compared against the performance of the Bessel scheme. The remainder of the paper is structured as follows. Section 2 describes the optical system, the custom FP ultrasound sensor, and the sensor biasing procedure. Section 3 describes the method and results of the sensitivity, directivity, and resolution tests. Section 4 discusses the findings, and Section 5 concludes the paper.

## 2. METHODS

### A. Interrogation System

**Fig. 1.**
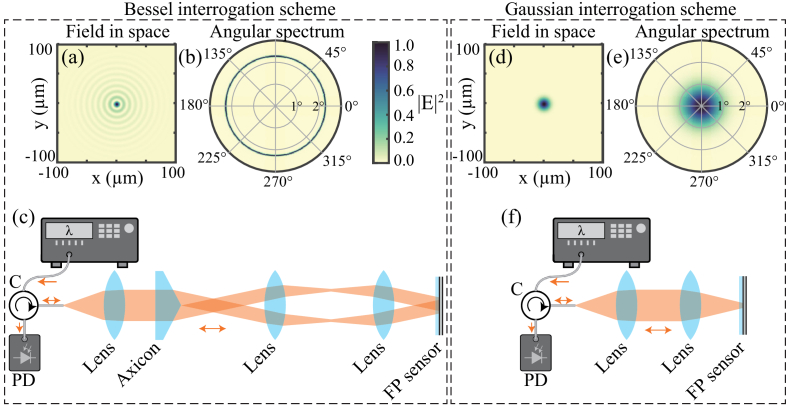
Experimental systems used to interrogate an FP ultrasound sensor using (a)–(c) a Bessel beam, showing (a) its 2D spatially resolved intensity profile in the focal plane of the interrogation system (coincident with the FP sensor’s first mirror), (b) its angular spectrum (the radial axis shows the angle between the plane wave components and the optical axis), (c) the interrogation system; (d)–(f) a Gaussian beam, showing the same as (a)–(c) but for the Gaussian beam. The beam profiles were computed using numerical models [25,26]. The angular spectra were calculated by Fourier transforming the complex spatial fields. The colorbar at the right side of (b) applies to all the color plots. The abbreviations PD and C stand for photodiode and circulator, respectively.

To test the concept of interrogating an FP ultrasound sensor using a Bessel beam, a beam design was chosen that would provide a core diameter of 29 µm, with a depth of focus of 54 mm [24]. To visualize the beam’s characteristics, its 2D cross-sectional spatial intensity profile and angular spectrum [20] were calculated [25] and are plotted in Figs. 1(a) and 1(b). As expected, the profile shows the focused core surrounded by several concentric rings of decreasing intensity. Meanwhile, the angular spectrum comprises a single ring centered on 2.29° with a full-width half-maximum (FWHM) of approximately 0.05°.

To enable interrogating an FP sensor with this beam, an experimental system was constructed. The system is shown in Fig. 1(c). Briefly, light from a wavelength-tunable continuous-wave laser (TSL-550, Santec) was delivered via a fiber optic circulator to a collimator (F220APC-1550, Thorlabs). The resulting collimated beam was transmitted through a 5° axicon (AX255-C, Thorlabs), forming a Bessel beam. This beam was relayed at normal incidence to an FP sensor by a 4f system formed by two plano-convex lenses (LA1509-C-ML, Thorlabs). In these conditions, light reflected by the sensor propagated back through the system to the circulator, which delivered it to a photodiode connected to a custom-made transimpedance amplifier [11]. Amplified signals from the photodiode, encoding the reflected light intensity, were measured using an oscilloscope (PicoScope 6403D, Pico Technology).

For comparison, experiments were performed using an FP sensor interrogated using a conventional Gaussian beam with a waist (intensity full width at ${1/e}^{2}$ ) of 50 µm. A spatial intensity profile and angular spectrum were computed and plotted in Figs. 1(d) and 1(e), respectively. As expected, both are Gaussian distributed. In the angular spectrum case, the distribution contains polar angles ranging from 0° to about 1.1° (intensity full width at ${1/e}^{2}$ ). To interrogate the FP sensor with this beam, the system was adapted, as shown in Fig. 1(f). Briefly, light exiting the circulator was collimated by a different collimator (F280-APC, Thorlabs), then focused at normal incidence onto the FP sensor using a lens (LA1509-C-ML, Thorlabs). As before, the reflected light propagated back through the optical system to the photodiode via the circulator.

### B. FP Ultrasound Sensor

To test the Bessel interrogation concept, an FP ultrasound sensor capable of attaining a high Q -factor, free of confounding factors due to optical imperfections, was required. To meet this requirement, a custom FP sensor with a thick planar spacer layer formed of high-quality optical grade fused silica was fabricated. Compared to typical FP ultrasound sensors with thin-film polymer spacers [11], this conferred two main advantages. First, the thicker cavity allowed for a higher maximum Q -factor. Second, the fused silica spacer afforded a high level of performance and confidence in a number of physical parameters including optical transparency [27], thickness uniformity [28], and surface smoothness [29]. A further property of the fused silica sensor was a lower compressibility. This led to a lower overall (ultrasonic) sensitivity. However, it did not limit our ability to fairly compare the different interrogation schemes.

To fabricate the FP sensor, a 500 µm thick fused silica layer was coated on both sides with 95% reflective dielectric mirrors. The mirrors were formed by alternating dielectric stacks of silicon dioxide ${SiO}_{2}$ and zirconium dioxide ( ${ZrO}_{2}$ ), with an optical thickness of a quarter of a chosen design wavelength of 1500 nm. To provide an acoustically matched backing layer, the sensor was affixed to a 2.5cm×2.5cm×2.5cm thick, anti-reflection coated-glass cube. For physical protection, the sensor was encapsulated in a 20 µm polyparachloroxylylene (PPXC) barrier layer [11].

### C. Selection of the Sensor Bias Wavelength

Preparing the system for ultrasonic sensing required selecting an appropriate interrogation laser power and bias wavelength as described above. The bias wavelength was selected by measuring an ITF and choosing the wavelength that maximized the value of the function [12]:

(1) $$\left|\frac{1}{ITF(\lambda )}\times \frac{dITF(\lambda )}{d\lambda }\right|,$$

where dITF(λ)/dλ was the derivative of the normalized ITF, which is proportional to the change in reflected power per unit pressure per unit interrogation power. Meanwhile, the term ITF(λ) was the normalized value of the ITF, which is inversely proportional to the ultrasonic sensitivity because it determines the usable interrogation power without saturating the photodiode. To explain briefly, when making ultrasonic measurements, the interrogation power was chosen such that the power received by the photodiode was equal to a predetermined (high) value limited by the photodiode’s saturation point. In these conditions, a lower ITF value led to a higher interrogation laser power, thus a higher sensitivity.

**Fig. 2.**
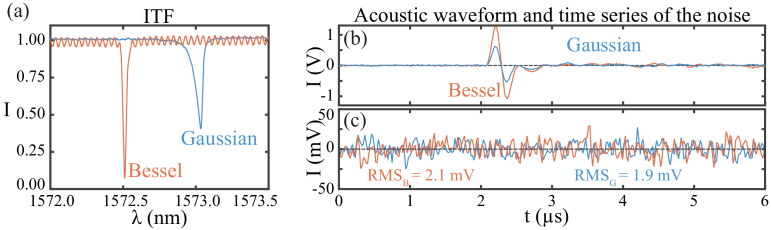
Sensitivity study. (a) ITFs of the FP sensor measured using the Bessel and Gaussian interrogation schemes. (b) Ultrasonic waveform measured in response to an ultrasonic plane wave using the Bessel and Gaussian interrogation schemes. The transducer was positioned a few millimeters from the sensor. (c) Time series of the reflectance without insonification measured to quantify the noise level of the systems. A 20 MHz first-order Butterworth low-pass filter was applied to the time series plotted in (b) and (c).

## 3. RESULTS

### A. On-Axis Sensitivity

To evaluate the relative on-axis ultrasonic sensitivity of the FP sensor interrogated using the Bessel scheme, ultrasonic waveforms obtained in response to a normally incident ultrasonic plane wave were compared to those obtained using a conventional Gaussian interrogation scheme.

First, measurements were made with the Gaussian scheme [Figs. 1(d)–1(f)]. Having set up the system, the sensor was insonified by an ultrasonic plane wave generated using a piezoelectric transducer (V380-SU, Olympus) with a central frequency of 3.5 MHz and a 67% bandwidth ( −6dB ). The ultrasonic waves were coupled to the sensor using water. To provide a plane wave, the transducer was driven by a pulse provided by a pulser-receiver (DPR 300, Altana BYK). To ensure a signal well within the linear working range of the FP sensor, the driving signal from the pulser was attenuated with a 20 dB attenuator.

To calculate the bias wavelength, the ITF was measured by recording the reflectance of the FP sensor while sweeping the interrogation wavelength. The ITF was plotted in Fig. 2(a) labeled Gaussian. The ITF was asymmetric along the wavelength axis, with visibility around 0.5, a maximum gradient of approximately $40\;{nm}^{-1}$ , and a Q -factor of $3\times 10^{4}$ . Taking into account the thickness and mirror reflectivity of the sensor, the low visibility and asymmetry observed were an expected consequence of the Gaussian beam providing a poor match to the planar cavity modes [16,19].

Having measured the ITF, the sensor was biased as described in Section 2.C, and an ultrasonic measurement was made. The resulting waveform was plotted in Fig. 2(b) labeled Gaussian. The waveform contained a single bipolar pulse with the expected shape and time of arrival given the known characteristics and location of the transducer. The pulse had a peak amplitude of 0.59 V, and the FWHM of the positive peak was 115 ns. To examine the noise level, the measurement was repeated with the pulse-receiver switched off. The resulting waveform is plotted in Fig. 2(c). The root mean square (RMS) of the noise voltage was 1.9 mV for a 20 MHz bandwidth, indicating a signal-to-noise ratio (SNR) of 311.

The process was repeated using the Bessel beam interrogation scheme [Figs. 1(a)–1(c)]. As before, an ITF was measured and plotted in Fig. 2(a) labeled Bessel. Compared to the ITF obtained with a Gaussian beam, this ITF is more symmetric, with a higher visibility of 0.8, a higher peak derivative of approximately $72\;{nm}^{-1}$ , and a higher Q -factor of $6\times 10^{4}$ . These changes are in line with expectations, given the better mode matching between the Bessel beam and the planar FP cavity [30]. The ITF obtained with the Bessel beam also exhibited a high-frequency low-visibility ripple, likely parasitic interference due to an optical cavity being formed between the FP’s first mirror and the first surface (air–glass interface) of the glass backing.

As above, having obtained the ITF, the sensor was biased, and an acoustic waveform was measured and plotted in Fig. 2(b) labeled Bessel. Like before, the ultrasonic waveform contained the expected bipolar pulse with a similar shape. However, as expected, the bipolar pulse had a larger amplitude, with a peak value of 1.22 V, indicating an increase of approximately 2.1 times in the on-axis ultrasonic sensitivity. This increase was slightly larger than the increase in the optical sensitivity, due to the fact that the higher visibility enabled the use of a higher laser power without saturating the photodiode. The FWHM of the positive peak was 114 ns (similar to the value for the Gaussian scheme). To check the noise level, a noise-only measurement was made and processed as above and plotted in Fig. 2(c). The noise level was 2.1 mV, similar to the value obtained with the Gaussian beam system, indicating an SNR of 581.

### B. Directivity

To examine the directivity of the FP ultrasound sensor interrogated with the Bessel and Gaussian schemes, the relative sensitivity as a function of the incident ultrasonic angle and frequency was measured for both the schemes.

**Fig. 3.**
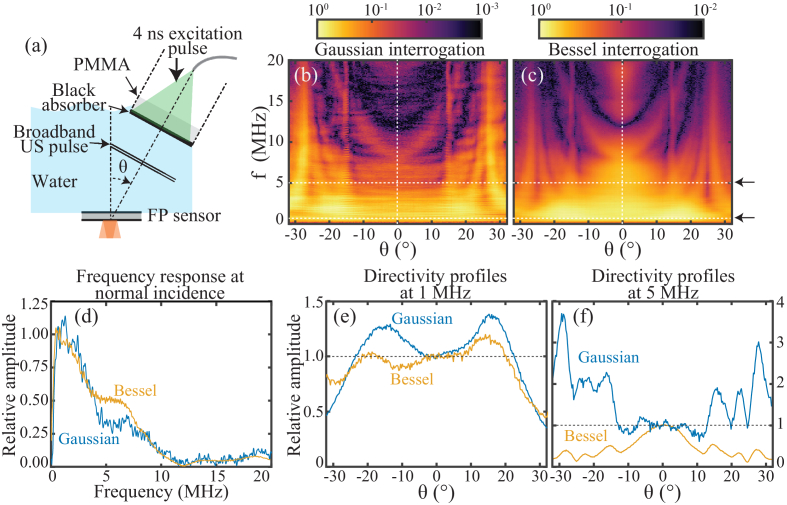
Directivity study. (a) Schematic of the system. (b) and (c) Relative sensitivity as a function of ultrasonic frequency and angle for the Gaussian (b) and Bessel (c) schemes. In (b) and (c), each vertical line of the heatmap is the absolute value of the Fourier transform of the ultrasonic waveform obtained at a specific angle. Waveforms were obtained with 100 times signal averaging. Prior to taking the Fourier transform, the averaged waveforms were windowed using a Tukey window with a width of 12 µs. (d) Relative sensitivity as a function of ultrasonic frequency at normal incidence. (e) and (f) Relative sensitivity as a function of angle at 1 MHz (e) and 10 MHz (f).

The setup [Fig. 3(a)] was similar to the one described previously [31]. Briefly, ultrasonic waveforms were measured in response to broadband ultrasonic plane waves incident upon the FP sensor at a range of different angles [31]. The plane waves were provided by a laser ultrasound source comprising a 5 mm thick polymethyl methacrylate (PMMA) disk with a 4 cm diameter, coated with a thin ( ≈10µm thick) layer of black spray paint. The paint layer was irradiated by 4 ns pulses of light provided by a pulsed laser system (Photosonus X, EKSPLA) delivered via a multimode fiber with a diameter of 1.5 mm and a numerical aperture of 0.22. The wavelength of the laser light was 1064 nm, and the pulse repetition rate was 100 Hz. The absorption of the light in the paint layer resulted in the generation of a plane wave via the photoacoustic effect. It is estimated that the plane wave contained detectable frequency components ranging from below 1 MHz up to at least 40 MHz. To control the plane wave’s angle of incidence upon the FP sensor, the laser ultrasound source was mounted on a motorized rotation stage (PRM1/MZ8, Thorlabs), whose rotation was controlled using custom control software [32,33]. To ensure that the rotation point was co-incident with the interrogation point, the rotation stage was aligned such that the ultrasound wave arrived at the interrogation point at the same time, independent of the angle.

As before, measurements were first made using the Gaussian interrogation scheme [Figs. 1(d)–1(f)]. The angular range of the measurements was from $-32^{\circ }$ to 32° with a step size of 0.25°. At each step, 100 waveforms were acquired for averaging. To visualize the sensor’s response as a function of ultrasonic frequency, the averaged waveforms were Fourier transformed. The resulting frequency spectra were stacked together to form a 2D dataset, which is plotted in Fig. 3(b), hereafter referred to as a directivity map.

Broadly speaking, the directivity map appears consistent with previous directivity data obtained using FP sensors made of thick glass cavities [34]. The map is visually angularly symmetric, with regions of high or low sensitivity, and regions in which the response is relatively uniform as a function of angle, e.g., at $\pm 10^{\circ }$ and ≤8MHz . Also evident, at certain angles, is a high sensitivity at all frequencies, e.g., at 15° and 27° [34].

The measurement was repeated using the Bessel interrogation scheme [Figs. 1(a)–1(c)]. The resulting directivity map was plotted in Fig. 3(c). As with the Gaussian scheme, the directivity map was angularly symmetric, with regions of relatively high and low sensitivity. Broadly speaking, the two maps look visually similar in terms of their spatial features.

To examine the data more closely, line profiles were extracted from the directivity maps at two orientations. First, a vertical line profile through the center was extracted, representing the frequency response at normal incidence. The resulting frequency responses were plotted in Fig. 3(d). The frequency responses are similar, both showing a smooth roll-off from low frequency to an apparent cut-off (around 10 MHz) as expected. Second, horizontal profiles were extracted at 1 and 5 MHz and plotted in Figs. 3(e) and 3(f). At 1 MHz, the angular responses of the two schemes were similar ( <50% variation). However, at 5 MHz, the responses clearly differ. Specifically, although the response is similar at normal incidence, as the angle increases, the sensitivity afforded by the Gaussian scheme increasingly exceeds that of the Bessel scheme. Referring back to the 2D directivity maps and their associated colorbars, it is evident that there is a region of generally lower sensitivity in the Bessel case, in the higher angle, high-frequency region of the plot. In other words, the Bessel beam was more directional at higher ultrasonic frequencies.

### C. Spatial Resolution in PAT Imaging

To evaluate the spatial resolution when performing PAT imaging, an experiment was designed to enable imaging of a resolution phantom using a single detection point on the FP sensor.

**Fig. 4.**
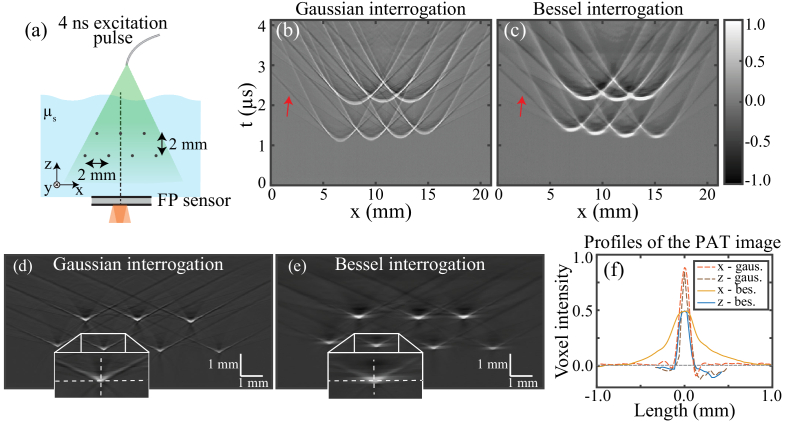
PAT imaging study. (a) Partial schematic of the experimental PAT setup. (b) and (c) Cross-sectional slices through the spatially resolved photoacoustic time series dataset measured using the (b) Gaussian and (c) Bessel schemes. (d) and (e) Cross-sectional slices through the corresponding PAT images. (f) x and z profiles through the center of one of the hairs, at the location indicated in the subfigures inset in (d) and (e).

The setup was similar to the one described previously [15]. Briefly, as shown in Fig. 4(a), a pulsed laser was used to excite photoacoustic waves in a phantom, which was mechanically scanned along two dimensions parallel to the plane of the FP sensor. Translating the phantom in this way, while detecting photoacoustic waves at one fixed position defined by the interrogation point on the sensor, allowed for emulating the detection of the waves at different positions as in a traditional FP-based PAT system in which the interrogation beam is scanned across the sensor surface [11].

The phantom was made of seven synthetic hairs with a diameter of approximately 60 µm. The hairs were oriented along the y axis, where the z axis was the axis normal to the FP sensor, as illustrated in Fig. 4(a). To generate the photoacoustic waves, the hairs were illuminated with a 4 ns pulsed laser with a wavelength of 1064 nm generated by a 100 Hz Photosonus X (EKSPLA). To homogenize the fluence and serve as a coupling medium for the ultrasound waves, the phantom was submerged in a liquid solution with a scattering coefficient of around $10\;{cm}^{-1}$ . The solution was made by diluting 20% intralipid in water with a ratio of 5%. To translate the phantom, it was mounted on two motorized linear stages (PT1-Z8 and MTS25/M-8, Thorlabs). While the phantom was scanned along the x axis, photoacoustic waveforms were measured every 25 µm across a total scan range of 2 cm. Meanwhile, the y axis location was varied in steps of 500 µm across a total scan range of 1 cm. The measured photoacoustic waveforms were stacked together along x and y to form a photoacoustic time series dataset. Using this dataset, a PAT image was reconstructed using a time reversal algorithm [35], assuming a sound speed of $1480\;{ms}^{-1}$ (water at 20°C).

Imaging was first performed while using the Gaussian interrogation scheme. A cross-sectional slice through the resulting photoacoustic time series dataset is plotted in Fig. 4(b). As expected, seven parabolic wavefronts were visible, one for each of the hairs, along with seven negative V-shaped artifacts [one of which is highlighted by the arrow in Fig. 4(b)]. The corresponding PAT image is plotted in Fig. 4(d). As expected, all seven hairs were visible in the image, along with V-shaped artifacts.

The process was repeated using the Bessel interrogation scheme. The resulting photoacoustic time series dataset is plotted in Fig. 4(c). Again, seven parabolic ultrasound wavefronts were seen with accompanying V-shaped artifacts. The corresponding PAT image is plotted in Fig. 4(e). As before, the seven hairs were visible with the V-shaped artifacts. However, as compared to the image obtained using the Gaussian beam, the hairs appeared visually more blurred out in the x direction.

To evaluate the spatial resolution, axial and lateral line profiles were taken through the center point of one of the hairs and plotted in Fig. 4(f). As expected, the profiles each contain a peak corresponding to the cross-section of the hair. The axial profiles were similar, with FWHMs of 80 µm and 112 µm for the Gaussian and Bessel schemes, respectively. However, the lateral profiles differed significantly, with the Bessel scheme yielding a significantly broader width (492 µm) compared to the Gaussian (112 µm). Beyond their widths, the Bessel scheme profiles have a lower peak intensity as compared to the Gaussian. Thus, in addition to the Bessel scheme providing a 4–5 times poorer lateral resolution, it also yielded lower image contrast (despite providing a higher on-axis ultrasonic sensitivity in Section 3.A).

## 4. DISCUSSION

A system was developed to test the concept of locally interrogating a planar FP ultrasound sensor using a Bessel beam. The principal motivation was that, compared to a conventional Gaussian beam, the Bessel beam might unlock a higher ultrasonic sensitivity while retaining a small acoustic element. The concept was tested by comparing the performance of a custom FP sensor interrogated by a Bessel beam with a 30 µm core and a Gaussian beam with a 50 µm waist. Two key comparisons were made, one of ultrasonic sensitivity and the other of the acoustic element size.

To compare the on-axis sensitivity, the SNRs obtained using the two beams were compared when making ultrasonic measurements. As anticipated, the Bessel beam provided a higher SNR than the Gaussian beam, indicating a higher on-axis ultrasonic sensitivity. The improvement, of approximately two times, can be attributed to two changes in the ITF afforded by the Bessel beam, namely a higher peak gradient and a higher visibility. These changes are consistent with a previous study in which an improved Q -factor was attained using a Bessel beam [30]. The changes can be attributed to the better mode matching between the Bessel beam and the planar FP cavity [30].

The acoustic element size was explored by measuring the ultrasonic directivity. Directivity is closely related to the element size in that larger elements become more directional due to spatial averaging, especially at higher frequencies [36]. At low frequencies, the Bessel scheme afforded a similar directivity to the Gaussian. However, at higher frequencies, the Bessel scheme proved more directional, suggesting more spatial averaging, thus a larger acoustic element size. To gain further insight, 3D PAT images were obtained, and the spatial resolution was estimated by examining line profiles through point-like features. The axial resolution was found to be similar for both the beams. However, the Bessel beam provided poorer lateral resolution, again suggesting a larger acoustic element [8].

Although the on-axis SNR in response to a 3.5 MHz ultrasonic pulse was higher for the Bessel scheme, the maximum PAT image intensity was higher for the Gaussian scheme [Fig. 4(f)]. This higher intensity is likely due to the Gaussian scheme’s less directional response, featuring higher sensitivity to several higher angles and frequencies (Fig. 3). As the PAT image reconstruction step effectively coherently sums contributions from all these angular and frequency components, the image intensity can readily be higher despite the lower on-axis sensitivity.

To properly evaluate the Bessel interrogation concept, the above experiments were performed using a custom FP ultrasound sensor. While most FP ultrasound sensors have a thin polymer spacer [2,11,12], this sensor had a thick spacer formed of fused silica. As explained, this provided confidence in a number of critical parameters, ultimately enabling a fair assessment of the Bessel scheme in a high Q -factor regime. It also led to two other non-critical differences from what would be expected with other FP sensors. First, the relatively low compressibility of fused silica reduced ultrasonic sensitivity. Second, due to its different acoustic properties, the PAT images had V-shaped artifacts [Figs. 4(b) and 4(c)]. This is thought to be due to ultrasonic waves coupling into the sensor at certain angles, then traveling along the sensor faster than their counterparts in the water. The resulting V-shaped wavefronts in the measured data led to V-shaped artifacts in PAT images. Although undesirable in other circumstances, neither of these issues diminished the present study. Indeed, the comparison between both the interrogation schemes is expected to apply equally well to all FP sensors, so long as they are capable of attaining a high enough Q -factor. In other words, for any planar FP sensor of similar thickness and optical quality, the same Bessel beam would be expected to provide the same relative (higher) ultrasonic sensitivity and (larger) element size compared to the same Gaussian beam.

The fact that the Bessel beam could yield a larger acoustic element is not entirely unexpected. While a Gaussian beam has a single well-defined focus, a Bessel beam’s core is surrounded by multiple rings, placing much of the beam’s energy outside of the core. Prior to this study, the effective size of a Bessel beam from the perspective of localized sensing using an FP ultrasound sensor was untested and thus unknown. To explore it here, as a first-order test, we selected a beam with a core size near to the focal size of a Gaussian beam typically used to interrogate an FP sensor. However, despite the small core size, our directivity and spatial resolution results both suggest the Bessel scheme yielded a larger acoustic element size. We conclude that the effective acoustic element size extends well beyond the core, i.e., at least some way into the region of the rings. These findings are consistent with evidence from other imaging technologies where Bessel beams provided lateral resolution considerably larger than the core size [37].

Although our Bessel beam yielded a larger element than our reference Gaussian, this may not be true for all such beams. For instance, a Bessel beam with a higher or lower depth of focus [25] would have more or less energy in its rings, likely influencing its effective acoustic element size. Further work could seek to quantitatively determine the relationship between the element size and the optical sensitivity for different Bessel beams to establish a more complete understanding of the broader landscape of all available combinations of sensitivity and element size. One way to pursue this could be to use a computational modeling approach, for example, combining relevant ultrasonic [36] and optical [16,19,30] models of FP ultrasound sensors to simulate relevant aspects of our experiments for a wide range of Bessel beams. Additionally, this study could also be performed for Gaussian beams (and other beams [12]) to better understand and compare the relation between sensitivity and element size for different beam types. To validate such studies, the experimental data presented in the present paper could provide a useful benchmark.

Comprehensively charting the landscape of available sensitivities and element sizes afforded by different Bessel (or other) beams could guide the design of FP ultrasound sensors for different applications. Across the many applications of FP ultrasound sensors, the relative importance of these parameters is application-dependent. For example, for deep tissue biomedical PAT, the top priority is often achieving the highest possible sensitivity at low ultrasonic frequencies [38]. Meanwhile, high-resolution superficial PAT [7] requires wide directivity at higher frequencies, requiring a balance of high sensitivity and directivity at a broad range of frequencies. Therefore, depending on the specific quantitative trade-offs afforded in terms of sensitivity and element size, different interrogation beams might be attractive for different applications.

## 5. CONCLUSION

The feasibility of using a Bessel beam to locally interrogate an FP ultrasound sensor was demonstrated. As compared to a conventional focused Gaussian beam of a size similar to the Bessel beam’s core, the Bessel beam afforded better mode-matching with the planar FP cavity, resulting in a higher optical Q -factor. As anticipated, this in turn yielded a higher on-axis ultrasonic sensitivity. However, ultrasonic directivity and photoacoustic spatial resolution measurements showed that the Bessel scheme also conferred a larger acoustic element size. Evidently, for an FP sensor interrogated by a Bessel beam, the effective acoustic element is not confined to the beam’s core but rather extends into the region of the rings. Here, this resulted in the selected Bessel beam attaining a higher on-axis sensitivity but only at the expense of a larger element. Further research could explore whether this would be the case for all Bessel beams and optimize the beam selection for different applications. By providing insight into the applicability of Bessel beams to FP ultrasound sensing, this work paves the way to a more complete understanding of the landscape of possible FP interrogation beams and the optimal design of systems based on FP ultrasound sensors for applications including PAT, ultrasonic imaging, and metrology.

## Data Availability

Data underlying the results presented in this paper are not publicly available at this time but may be obtained from the authors upon reasonable request.
